# Can FoCUS Speed Up the Management of Acute Coronary Syndrome in the Emergency Department?

**DOI:** 10.3390/medicina62061013

**Published:** 2026-05-23

**Authors:** Melina Karaolia, Sofia Bezati, Katerina Papasolomou, Estela Kiouri, Christos Verras, Effie Polyzogopoulou

**Affiliations:** Department of Emergency Medicine, Attikon University Hospital, Rimini 1, 12462 Chaidari, Greece; melinakaraolia@gmail.com (M.K.); sofiabezati@gmail.com (S.B.); pap_kate@outlook.com (K.P.); estelakiouri@hotmail.com (E.K.); christos.verras@gmail.com (C.V.)

**Keywords:** focused cardiac ultrasound, FoCUS, acute coronary syndrome, chest pain, emergency department

## Abstract

Focused Cardiac Ultrasound (FoCUS) is a targeted bedside imaging modality with an established role in the management of critically ill patients. Acute Coronary Syndrome (ACS) is a common cause of presentation to the Emergency Department (ED), and although electrocardiography (ECG) and cardiac biomarkers are the cornerstones for its diagnosis, FoCUS may facilitate diagnostic evaluation and disposition of patients in different levels of care. Initially, FoCUS plays a crucial diagnostic role through the identification of Regional Wall Motion Abnormalities (RWMAs), enabling direct visualization of the ischemic region and corroboration of ECG findings. Moreover, in patients with ACS complicated by cardiogenic shock, FoCUS is indispensable for determining the extent of ischemia and detecting mechanical complications, including ventricular septal or free wall rupture, or papillary muscle rupture. Likewise, FoCUS aids in the differential diagnosis of patients with ECG abnormalities mimicking ACS. This comprehensive review synthesizes the most recent evidence on the role of FoCUS in accelerating the management of patients with ACS presenting to the ED.

## 1. Introduction

Chest pain is the primary non-traumatic cause of presentation to the Emergency Department (ED). Although Acute Coronary Syndrome (ACS) will be detected in a small proportion of patients with chest pain, ranging from >5% to 13% [1,2], it carries significant morbidity and mortality, demanding appropriate intervention in a timely manner. Early identification of high-risk patients may improve patient outcomes and optimize the use of valuable resources [1,2,3].

Electrocardiography (ECG) and evaluation of cardiac Troponin (cTn) levels are the cornerstones in the diagnostic evaluation of patients with suspected ACS and are embedded in diagnostic and risk-stratification pathways [4]. However, both can be inconclusive and exhibit low sensitivity in certain clinical scenarios, mainly in patients with Unstable Angina (UA) or Non-ST-segment elevation Myocardial Infarction (NSTEMI) [2]. Focused Cardiac Ultrasound (FoCUS) is a valuable bedside tool providing critical clinical information, which, in combination with ECG and biomarkers, may assist and expedite diagnosis. This narrative review will emphasize the contribution of FoCUS in complementing diagnostic workup and facilitating management of patients with ACS in the ED. For this purpose, we performed a literature review in the PubMed database using the following keywords: “chest pain”, “Focused Cardiac Ultrasound”, “Acute Coronary Syndrome”, “Regional Wall Motion Abnormalities”, “ACS complications” and “Emergency Department”.

## 2. What Are the Major Applications of FoCUS in Chest Pain Evaluation?

Point-of-care ultrasound is a non-invasive, low-cost, and time-effective modality introduced as a promising tool in the ED, during all stages of clinical evaluation and management of critically ill patients, from diagnosis to treatment and monitoring [5]. The technological evolution of smaller and portable devices has enabled rapid evaluation at the bedside. Likewise, in patients suspected of ACS, Focused Cardiac Ultrasound (FoCUS) may accelerate clinical assessment and decision-making by providing rapid global cardiac and hemodynamic evaluation. It should be highlighted that “FoCUS is defined as a point-of-care cardiac ultrasound examination, performed according to a standardized, but restricted, scanning protocol, as an extension of the clinical examination”. Its application is indicated in various clinical scenarios such as patients with circulatory compromise/shock, cardiac arrest, chest pain/dyspnea, chest/cardiac trauma, respiratory compromise and syncope/presyncope [6].

A distinguishing feature between FoCUS and comprehensive echocardiography is that FoCUS is performed by the treating physician as a means to complete clinical evaluation. FoCUS aims to acquire images that may provide additional information regarding the underlying pathology in order to narrow differential diagnosis, to rule out or rule in specific clinical pathologies according to the clinical setting, and to tailor further management or trigger additional workup [6,7,8]. Basic FoCUS includes qualitative estimation of left- and right-ventricular function and dimensions, pericardial effusion, as well as assessment of volume status and gross assessment of heart valves. Advanced FoCUS shares the same underlying philosophy as basic FoCUS but differs in its complexity as it is performed by more experienced operators using multiple ultrasound modalities, such as color Doppler, pulse-wave Doppler and M-mode [8].

Firstly, one of the major applications of FoCUS is its adjuvant role in the diagnosis of ACS, allowing the visualization of structural and functional abnormalities. Moreover, in patients with ACS complicated by impaired cardiac contractility, FoCUS can effectively assess global left and right-ventricular systolic function and promptly identify severe mechanical complications, including papillary muscle rupture (PMR), ventricular free wall rupture (FWR), or aneurysm or ventricular septal rupture (VSD) [3,9,10]. It should be emphasized that FoCUS has a complementary role in the assessment of patients with chest pain suspected of ACS, with the potential to identify features suggestive of acute ischemia. Yet, it should not be used as a stand-alone test to rule out ACS in the absence of pathological findings.

Furthermore, FoCUS may aid in the differential diagnosis of conditions mimicking ACS, such as pericarditis, cardiac tamponade, acute pulmonary embolism and Takotsubo cardiomyopathy.

## 3. What Are the Major Applications of FoCUS in ACS?

Several studies highlight that the addition of cardiac ultrasound in the initial evaluation of moderate to high-risk patients with undifferentiated chest pain and an ECG negative for STEMI enables the detection of RWMAs or the early diagnosis of the ACS complications [3,9]. In addition, in patients with STEMI presenting with hemodynamic instability, FoCUS may identify complications demanding mechanical support prior to coronary angiography or surgical management [11].

### 3.1. Regional Wall Motion Abnormalities

Its primary advantage is the early detection of regional wall motion abnormalities (RWMAs), which commonly develop before the appearance of electrocardiographic and biomarker abnormalities, even before the onset of chest pain [3,9].

RWMAs refer to the abnormal myocardial movement involving regions of the wall of the left ventricle, where impaired contraction and relaxation of the heart muscle are seen using ultrasound imaging. Motion abnormalities can be described as hypokinesis if there is a decrease in wall motion contractility, dyskinesis if there is paradoxical motion during systole, or akinesis in the absence of regional contractility [12].

**Figure 1:**
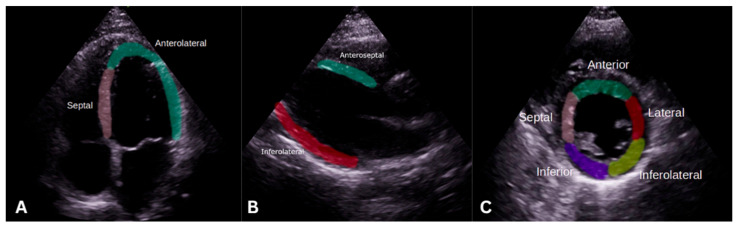
Illustrating the appropriate segments for the detection of regional wall motion abnormalities (RWMAs). (**A**) Apical-4-chamber view illustrating the anterolateral and septal regions of the left ventricle. (**B**) Parasternal long axis view illustrating the anteroseptal and inferolateral regions of the left ventricle. (**C**) Parasternal short axis view illustrating the anterior, lateral, inferolateral, inferior and septal segments of the left ventricle (image courtesy of E. Polyzogopoulou).

RWMAs are most commonly induced by ischemia and can present very early in the course of an ischemic event, even within seconds, and precede the findings of ECG changes and pain [13]. False-positive findings may result in the context of other pathologies, namely a prior infarction, focal myocarditis, Takotsubo cardiomyopathy, bundle branch block or prior surgery, especially valvular [14]. Τhe American Society of Echocardiography uses a 17-segment model for wall motion abnormalities [15]. However, in the emergency setting, a more condensed model is used, which includes five basic regions: lateral, anterior, septal, inferior and inferolateral (Figure 1, Supplemental Video S1). The location of an RWMA highly correlates with the ECG findings, both in ST-elevation Myocardial Infarction (STEMI) and NSTEMI, and can corroborate them in recognizing the culprit coronary artery, creating a “coronary pattern”, although there is significant overlap in the supply of the different areas [16].

FoCUS, when performed by properly trained emergency physicians, has a high sensitivity in detecting wall motion abnormalities comparable to that of a comprehensive echocardiography [17,18]. In a prospective study involving patients with suspected NSTEMI, properly trained emergency physicians performing FoCUS, when compared to comprehensive echo, were found to have a sensitivity of 76.9% and a specificity of 92.1% in detecting RWMAs [18]. Moreover, in a retrospective observational study including patients with chest pain in the ED, FoCUS showed a sensitivity of 94%, specificity 35% and overall accuracy of 78%, compared to a comprehensive echocardiography for the detection of RWMAs. In practice, FoCUS identified RWMAs in 87% of patients with Occlusion Myocardial Infarction (OMI) [19].

The presence of RWMAs is a strong independent predictor of ACS [20,21] and could identify patients who would benefit from expedited care (catheterization), especially patients who do not fulfill STEMI criteria but have acute occlusive coronary artery disease. The use of FoCUS for identifying WMA has a high specificity in detecting patients with acute coronary occlusion when applied in high-risk patients with acute chest pain [11,21], and their identification should trigger the performance of a comprehensive echocardiography for their confirmation [6,22]. In these patients, both diagnosis and time to revascularization may be expedited [23]. However, due to low sensitivity, the absence of RWMAs cannot be used to rule out ACS in these patients [11].

Moreover, incorporating FoCUS findings in risk scores such as the HEART risk score enhances diagnostic accuracy by increasing sensitivity, thus resulting in improved risk stratification [20]. A post hoc analysis of the EPIC-ACS study demonstrated that RWMAs could independently predict the presence of obstructive coronary artery disease and enable decision-making in patients presenting with chest pain to the ED and ECG and biomarker testing are inconclusive [24]. Yet, RWMAs may have low sensitivity in cases of small infarct size, non-transmural ischemia, as well as poor acoustic windows [21].

There is a growing body of evidence that highlights the role of WMA in the management of patients at high risk for ACS, but as most studies are retrospective or single-center studies, with a limited number of patients, further studies are still needed to validate the results and show concrete patient outcomes that will allow the incorporation of RWMAs in diagnostic algorithms.

### 3.2. Assessment of Global Cardiac Function

Global cardiac systolic function refers to the ability of the heart to act as a pump, and is assessed using cardiac ultrasound, by estimating Ejection Fraction (EF), a measurement evaluating the proportion of blood exiting the left ventricle in each cardiac cycle [25].

In the ED, the most popular method for estimating cardiac systolic function is the visual method (or “eyeballing”), where the operator estimates left-ventricular EF (LVEF) by observing the endocardium excursion and myocardium thickening of the left ventricle, as well as the movement of the mitral valve leaflet, using multiple cardiac views. EF can be characterized as normal or reduced [10]. In a meta-analysis of nine studies, FoCUS increased sensitivity and specificity of bedside clinical assessment for LV dysfunction from 43% to 84% and from 81% to 89% for sensitivity and specificity, respectively [26]. FoCUS, when applied by emergency physicians with core training in cardiac ultrasound, can be comparably effective as a comprehensive echocardiography [27].

In patients with suspected ACS, the assessment of EF and the overall evaluation of systolic function of the left ventricle may facilitate patient management by helping clinicians consider the need for further diagnostic workup or treatment [10]. Moreover, assessing global systolic dysfunction has prognostic value, as patients with significantly impaired EF exhibit higher short and long-term mortality [28].

Visual estimation of LV cardiac function in the ED is an essential and rapid examination. Nonetheless, it is a subjective method and its accuracy is affected by the experience of the operator [29]. Recently, a number of AI-assisted methods [30,31] have emerged showing promising results in enhancing diagnostic accuracy and reducing operator dependence. Moreover, standardized algorithmic interpretation can reduce variability between observers, which is frequently encountered in visual estimation [32]. A systematic review of observational studies in various clinical settings reported high diagnostic accuracy of AI-assisted FoCUS in estimating LVEF, as demonstrated by an Area Under the Curve of 0.85–0.98, suggesting its possible use as an adjunctive bedside tool for rapid assessment of global cardiac function [33].

## 4. What Are the Major Applications of FoCUS in ACS Complicated by HF and Cardiogenic Shock?

### 4.1. Severe Left-Ventricular Dysfunction

Acute Heart Failure (AHF) is frequent in ACS, and their combination can result in a poor prognosis. Patients with severely impaired LV function are in danger of developing cardiogenic shock with high in-hospital mortality.

In patients presenting with chest pain and signs of AHF, FoCUS is mandatory so as to identify the presence of severely reduced LVEF [34] (Video S2 in the Supplementary Materials), as described in the previous section. A meta-analysis including eight studies on the diagnostic accuracy of FoCUS for the identification of cardiogenic shock reports a pooled sensitivity of 86.1% and a pooled specificity of 95.8% [35]. In patients with ACS presenting with dyspnea, integration of lung ultrasound in the diagnostic approach may allow for a more comprehensive assessment. Identification of B-lines and/or pleural effusion along with impaired cardiac contractility may suggest the presence of AHF precipitated by an acute ischemic event [36].

Furthermore, in patients with or without clinical signs of overt cardiogenic shock, LV outflow track–Velocity Time Interval (LVOT-VTI) can be utilized to assess hemodynamic status and facilitate treatment decisions [37]. LVOT-VTI is a simple echocardiographic measurement, included in advanced FoCUS, using pulse-wave Doppler to estimate stroke volume and cardiac output. It is obtained using a five-chamber view at the level of the LV outflow tract, tracing the area using echocardiography software. LVOT-VTI values between 18 and 22 cm are indicative of adequate stroke volume, while lower values indicate impaired forward flow. Its application may facilitate early recognition of low-flow states, thus predicting deterioration, even if LVEF is preserved. LVOT-VTI values can guide appropriate administration of fluids or vasopressors and monitor response to treatment, especially when combined with lung ultrasound and assessment of the inferior vena cava [36]. Moreover, in patients with AHF, LVOT-VTI may be used as a risk stratification measurement, as low LVOT-VTI values are associated with increased mortality [36]. Measurement of LVOT-VTI is simple and, with the appropriate training, can be easily utilized by emergency physicians at the bedside [38].

Advanced FoCUS can also provide evidence of diastolic dysfunction, which can be present in patients with ACS and signs of AHF, regardless of LVEF. Diastolic dysfunction may be the first manifestation of an acute ischemic event, and precede ECG changes and systolic dysfunction [39].

The assessment of E/a ratio has high diagnostic accuracy for the detection of AHF in symptomatic patients [40]. Additionally, significant diastolic dysfunction in patients with acute MI is associated with more extensive myocardial damage and worse long-term myocardial outcome [41].

Both measurements provide useful insights into patients with severe LV dysfunction, but are considered advanced and are not included in the basic FoCUS examination [7,22,42,43]. In a prospective observational study examining the feasibility of performing these measurements in the ED, the average time for assessing both LVOT-VTI and LV diastolic dysfunction was more than 6 min, and the timing, as well as success, in obtaining this information from appropriate images, was dependent on the operator’s experience [44]. Thus, their application in the appropriate clinical context may aid in the early recognition of patients with Acute Myocardial Infarction (AMI) in need of mechanical circulatory support and/or early revascularization [34].

### 4.2. Right-Ventricular Failure

Right-ventricular failure due to right-ventricular Myocardial Infarction (RVMI) occurs most commonly as a result of inferior wall MI, presenting in more than 50% of cases. RVMI may rarely present in isolation or in association with anterior wall MI. RV failure may also occur in the context of acute LV failure [45]. In ACS complicated by RV failure, hemodynamic derangement is common and patients are preload sensitive and can develop profound hypotension, as well as electrical complications [46]. Assessment of RV function using FoCUS is invaluable for prompt diagnosis of RV failure, for guiding fluid resuscitation and the use of inotropes, as well as for monitoring response to treatment [47].

Visual assessment of RV function is paramount for identifying RV failure. All cardiac views should be obtained, but A4C and subxiphoid views are particularly important to assess the size of the right ventricle compared to the left ventricle at the end diastole and evaluate for RV dilatation, a common finding in RV failure. Values of the RV/LV ratio more than 0.6 signify RV dysfunction, with a ratio more than 1 suggesting severe RV dysfunction [47].

In cases of RV wall ischemia, regional wall motion abnormalities of the right ventricle can be visualized as hypokinesia or akinesia of the RV free wall, including the apex in the majority of cases. Due to severe dilation of the RV wall, D-sign may be produced in the Parasternal short-axis view and abnormal movement of the interventricular septum may be present [48]. Quantitative assessment of RV function can complement visual assessment and improve diagnostic accuracy for RV failure. Tricuspid Annular Plane Systolic Excursion (TAPSE) is a parameter, included in advanced FoCUS, that utilizes M-Mode to assess longitudinal RV systolic function in the A4C view. TAPSE is considered a reliable measure of RV function and correlates well with RV Ejection Fraction. A cutoff value of <17 mm signifies systolic dysfunction. Emergency physicians seem to perform TAPSE measurements competently, although most studies are performed in the setting of pulmonary embolism [49].

Moreover, FoCUS can be used to assess for the presence of tricuspid valve regurgitation, a common finding associated with RV failure after Myocardial Infarction, or to detect associated mechanical complications [45].

### 4.3. Mechanical Complications

#### 4.3.1. Papillary Muscle Rupture

Although papillary muscle rupture (PMR) is considered an extremely rare complication of AMI, occurring in up to 0.3% of cases, it is still associated with 40% in-hospital mortality, including patients undergoing surgery [50,51]. Therefore, a high level of clinical suspicion is needed to guarantee early identification and provision of hemodynamic support and invasive therapy. Cardiac auscultation is inconclusive as the characteristic systolic murmur is often absent due to the high left atrial pressures [52]. Thus, in patients with suspected subacute AMI and signs of acute pulmonary edema or cardiogenic shock, FoCUS is a valuable diagnostic tool for bedside evaluation of the presence of complete or partial PMR [53].

Sonographic findings of PMR include the presence of a mobile echogenic structure, partially connected or detached from the LV wall, moving irregularly within the LV and prolapsing to the left atrium, proportionally to the degree of rupture. Mitral valve leaflet prolapse, severe mitral regurgitation and LV motion wall abnormalities often co-exist [53,54]. Acute ischemic MR may also result from imminent PMR or from the displacement of the papillary muscle due to left-ventricular RWMAs, causing asymmetric leaflet tethering [55].

**Figure 2:**
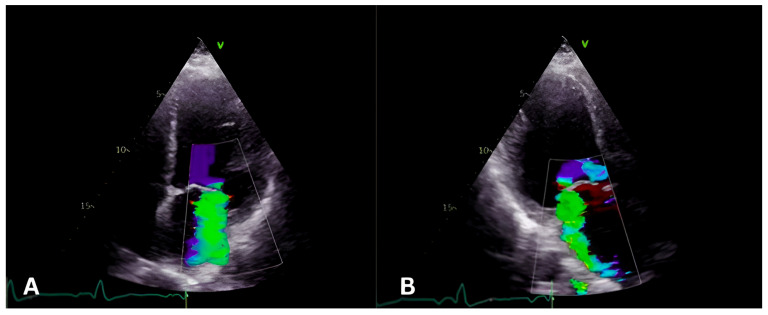
Severe acute mitral regurgitation with color Doppler; (**A**) apical-4-chamber view; (**B**) apical-3-chamber view (image courtesy of E. Kiouri).

Advanced FoCUS is an essential imaging method for the evaluation of acute MR as it enables the assessment of the mitral anatomy, the identification of the underlying mechanism and the estimation of MR severity. The exam should also include an evaluation with color Doppler. A regurgitant flow occupying >50% of the left atrium or an eccentric flow extending along the atrial wall indicates severe MR (Figure 2A,B and Videos S3 and S4 in the Supplementary Materials). The proximal flow convergence (PFC) and the vena contracta width (VCW) are two additional measurements for quantifying MR severity [55], yet their use may not be applicable in the case of PMR, where MR is mostly severe [53]. Furthermore, the size and function of the left cardiac chambers are evaluated. In acute MR, LV and LA dimensions are usually normal due to insufficient time for compensatory changes. LV contractility may present hyperdynamic [56].

The recent literature on the diagnostic utility of FoCUS reports a satisfactory diagnostic accuracy for the detection of MR (sensitivity of 76–100% and specificity of 87–100%) [26] and for the detection of PMR (sensitivity of 53.8%, specificity of 100%, overall diagnostic accuracy of 99,9%) [57]. Moreover, its ability to identify a flail leaflet has yielded 88% sensitivity [58].

#### 4.3.2. Ventricular Septal Rupture

Ventricular septal rupture (VSR) complicates AMI in 0.3% of cases [59] and the incidence is higher in patients with anterior AMI [60,61,62].

**Figure 3:**
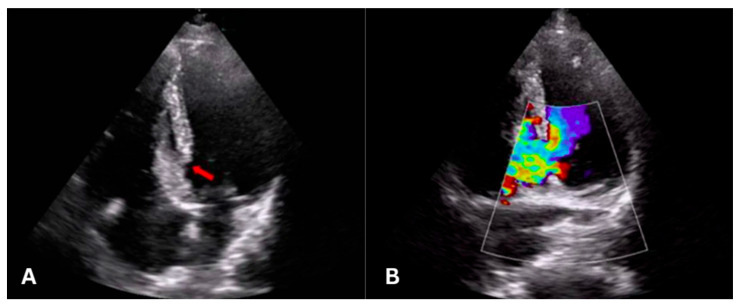
Ventricular septal rupture: (**A**) apical-4-chamber view, B-mode imaging illustrating the defect (red arrow) within the basal interventricular septum; (**B**) apical-4-chamber view, color Doppler depicting the left-to-right shunt (image courtesy of E. Polyzogopoulou).

In anterior AMI, VSR is typically simple and most commonly located at the apex of the heart. On the other hand, in inferior AMI, septal rupture has a complex morphology, involves the basal posteroinferior portion of the interventricular septum (Figure 3A,B and Video S5a,b in the Supplementary Materials), and when accompanied by right-ventricular (RV) involvement, represents an important predictor of adverse clinical outcomes [63,64].

FoCUS, enhanced by advanced modalities (color Doppler), represents a fundamental diagnostic tool for identifying the location, morphology and the severity of VSR in patients with AMI and dyspnoea. Moreover, its use serves in the differential diagnosis of other complications or causes of cardiogenic shock following AMI [61]. RV function should also be evaluated, as RV dilatation is a key finding, suggesting the need for urgent mechanical circulatory support as a bridge to surgery [59,61].

In an observational study including 18 patients with AMI and a newly detected murmur, B-mode enabled direct visualization of the septal rupture in 10 patients and correctly excluded the presence of a ventricular septal defect in the remaining cases. The A4C view and the subxiphoid view were the most useful echocardiographic windows for detecting the lesion [65]. However, the direct visualization of the septal defect using B-mode alone is often limited. In a study including 43 patients with confirmed VSR, direct visualization of the defect was achieved in only 40% of cases. In contrast, color Doppler proved to be a highly reliable diagnostic modality, demonstrating sensitivity and specificity approaching 100% both for detecting septal rupture and for differentiating it from acute severe mitral regurgitation [66]. Furthermore, in a retrospective study of patients with AMI who underwent emergency advanced FoCUS, the diagnosis of ventricular septal rupture was established in 93.3% of cases, highlighting the accuracy of FoCUS in the emergency setting [67].

Several studies have demonstrated that color Doppler is superior to B-mode, as it not only facilitates the detection of septal rupture but also allows precise localization of the septal defect, thereby contributing to the selection of the most appropriate therapeutic and surgical management strategy [68,69].

#### 4.3.3. Free LV Rupture

**Figure 4:**
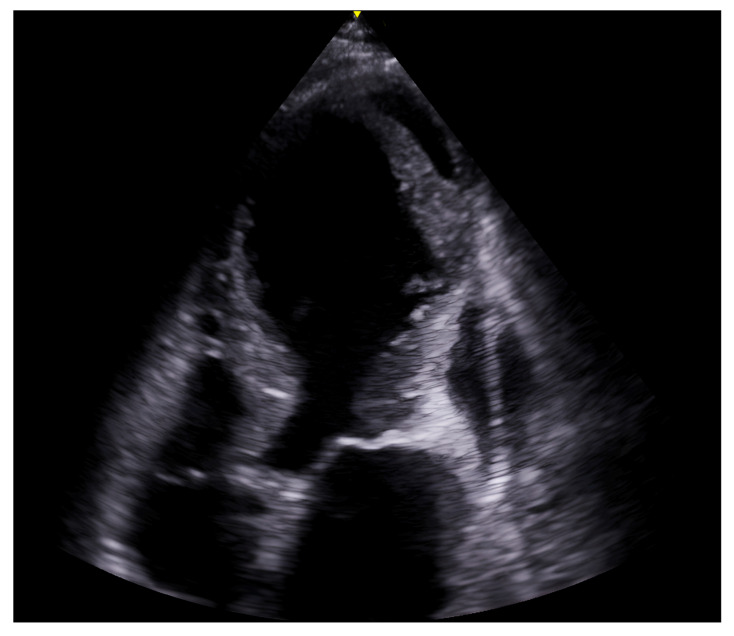
Apical-4-chamber view illustrating free left-ventricular wall rupture (image courtesy of E. Polyzogopoulou).

The acute form of rupture (blowout) typically results in sudden hemodynamic collapse or cardiac arrest and rarely allows sufficient time for therapeutic intervention. In contrast, the subacute form (oozing) (Figure 4 and Video S6 in the Supplementary Materials) progresses with slower bleeding, providing a brief but critical window for diagnosis with FoCUS and for the implementation of immediate life-saving interventions. The clinical manifestations of LV free wall rupture are primarily determined by the rate of pericardial bleeding and the subsequent development of cardiac tamponade [70].

The mean time to diagnosis of hemopericardium in the ED using FoCUS has been reported to be approximately ten minutes [71]. Pericardial effusion represents the most frequent echocardiographic finding in patients with subacute LV free wall rupture, although it may also be observed in patients with AMI without rupture [67,72]. Its presence—particularly when accompanied by echo-dense masses, myocardial wall defects, or signs of cardiac tamponade—in combination with clinical findings and the assessment of non-infarcted myocardial wall motion, significantly improves diagnostic accuracy [72].

More specifically, the presence of a pericardial effusion > 5 mm demonstrates 100% sensitivity for the diagnosis of subacute ventricular wall rupture. In addition, the presence of cardiac tamponade, high-density intrapericardial echoes, or compression of the right atrial or right-ventricular wall shows high diagnostic sensitivity (>70%) and specificity (>90%). Although each finding individually is associated with a relatively high rate of false-positive results (>20%), the combined assessment of clinical and echocardiographic parameters significantly improves diagnostic accuracy and reduces false-positive rates (<10%). Subacute rupture requires immediate surgical management, with reported survival rates of 76% following surgery and long-term survival of 48.5% [73].

Finally, a retrospective cohort study demonstrated that in-hospital mortality in patients with free LV wall rupture is not influenced by the time of hospital presentation. Instead, the most important prognostic factor is the hemodynamic status at admission, particularly the presence of cardiac arrest or cardiogenic shock, highlighting the importance of early diagnosis and prompt management of hemodynamic instability in the ED [70].

Table 1 summarizes possible key findings that may be identified in the context of ACS.

## 5. What Are the Major Applications of FoCUS in ACS Complicated by Cardiac Arrest?

FoCUS in ACS complicated by cardiac arrest facilitates accurate rhythm assessment, identification of other reversible causes and evaluation of cardiac activity to guide resuscitative efforts [74,75]. It enables differentiation between true and pseudo-PEA, which has significant prognostic and therapeutic implications. Additionally, FoCUS detects tamponade and life-threatening mechanical complications, such as free wall or papillary muscle rupture. It provides real-time procedural guidance and supports critical clinical decision-making and prognostication [76]. Despite its value, FoCUS should be used as an adjunct and not as a sole criterion for termination of resuscitation [77].

## 6. Can FoCUS Differentiate Other Mimickers of Chest Pain?

### 6.1. Takotsubo Cardiomyopathy

Takotsubo or stress-induced cardiomyopathy has a clinical and electrocardiographic presentation sometimes indistinguishable from an acute coronary syndrome. Absence of obstructed coronary arteries in coronary angiography is required for a definite diagnosis [78].

**Figure 5:**
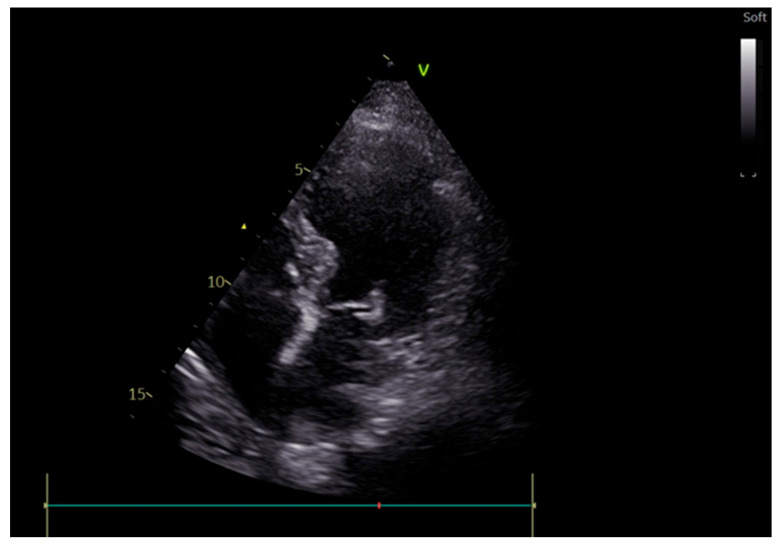
Patient with Takotsubo cardiomyopathy, apical-4-chamber view illustrating apical ballooning (image courtesy of E. Kiouri).

Nonetheless, in conjunction with other diagnostic criteria, there are specific echocardiographic findings suggestive of Takotsubo cardiomyopathy [79]. Takotsubo exhibits a characteristic pattern regarding wall motion abnormalities that extends beyond the typical coronary artery distribution. In its typical presentation, the LV exhibits an apical ballooning pattern (Figure 5), with apical dyskinesia or akinesia and hyperkinesia of the basal segment, demonstrated primarily in the PSL and A4C views. This presentation resembles a Japanese octopus trap, taking the name “Takotsubo” (Video S7 in the Supplementary Materials). Uncommonly, other patterns appear that involve mid-ventricular ballooning (14.6%), basal pattern or reverse takotsubo, where there is dyskinesia of the basal segments and hypercontraction of the apical region (2.2%) and focal pattern, where motion abnormality occurs in a small region of the LV wall (1.5%) [79,80]. Additionally, Ejection Fraction is usually reduced and patients with Takotsubo cardiomyopathy tend to present with a lower LVEF than patients with AMI. In some cases, right-ventricular dysfunction may be present, resulting in biventricular ballooning [80,81].

Although coronary angiography remains the exam of choice to reach a definite diagnosis, FoCUS can be used as an adjunct tool for the early identification of this entity in patients with a high suspicion of having Takotsubo cardiomyopathy.

### 6.2. Acute Pericarditis

**Figure 6:**
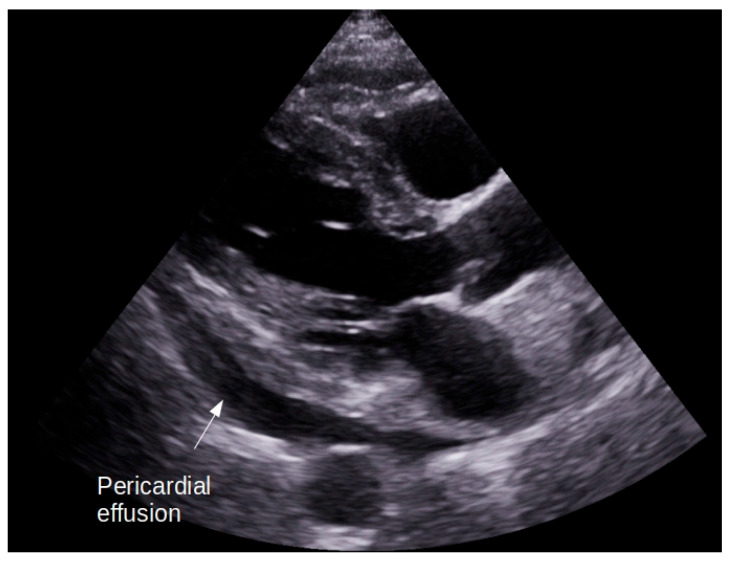
Parasternal long-axis view with pericardial effusion (image courtesy of E. Polyzogopoulou).

FoCUS is the first-line imaging modality included in the diagnostic workup of acute pericarditis, as a new or worsening pericardial effusion may be observed in up to 60% of cases [82]. The size of the pericardial effusion is estimated by measuring the end-diastolic distance of the echo-free space between the epicardium and the parietal pericardium [83] (Figure 6). Emergency physicians can detect pericardial effusion with high diagnostic accuracy, demonstrating 96% sensitivity, 98% specificity, and an overall diagnostic accuracy of 97.5% [84]. In another study, FoCUS performed at bedside showed moderate diagnostic accuracy; 64% sensitivity and 91.2% specificity, yet the high negative predictive value (93.6%) suggests that bedside FoCUS could be used for the exclusion of acute pericarditis in the appropriate clinical context [85]. Similar findings have been reported in studies in which emergency medicine trainees, following a short training program in FoCUS, detected pericardial effusion with 60% sensitivity, 100% specificity, a positive predictive value of 100%, and a negative predictive value of 97.9% [86].

Regarding its role in the differential diagnosis of patients with suspected ACS, a relevant study reported that the implementation of an ultrasound-based protocol led to a modification of the initial clinical diagnosis or of the therapeutic management in 14% of cases, while in one case the diagnosis was revised to pericarditis, thereby avoiding the need for coronary angiography [87]. Therefore, POCUS represents a valuable complementary tool for the rapid and targeted evaluation of patients presenting with chest pain to the ED.

### 6.3. Acute Pulmonary Embolism

Differentiating between patients with pulmonary embolism (PE) and ACS in the ED can be challenging. Up to one-third of patients with acute pulmonary embolism can present with symptoms, ECG, and laboratory findings suggestive of ACS [88].

**Figure 7:**
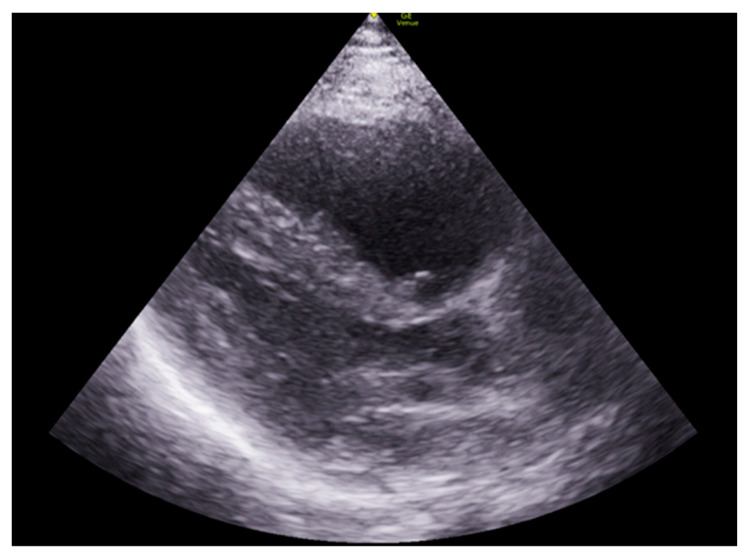
Parasternal long axis view depicting dilation of the right ventricle (image courtesy of S. Bezati).

**Figure 8:**
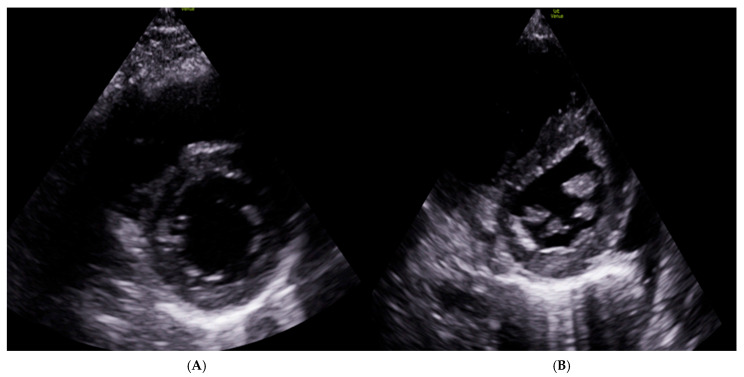
Parasternal short axis view; (**A**) normal morphology of the interventricular septum; (**B**) D-sign in the context of acute pulmonary embolism (image courtesy of E. Polyzogopoulou).

The presence of abnormal septal movement, RV dilation (Figure 7), D-sign (Figure 8B), McConnell’s Sign (Video S8 in the Supplementary Materials), RV hypokinesis, decreased TAPSE, Tricuspid Regurgitation and RV thrombus are all findings that suggest right heart strain and support the diagnosis of acute PE. All findings have very low sensitivity in detecting PE, but high specificity, with D-sign, McConnell’s sign and visible right-ventricular thrombus showing specificity above 95% [89].

However, in patients with RVMI, differentiation from PE can be extremely challenging, as both conditions exhibit signs of RV strain in cardiac ultrasound studies [90]. In RVMI, wall motion abnormalities of the posterior and inferior segments of the left ventricle are frequently present, and LVEF may be compromised. Moreover, the free wall of the right ventricle is diffusely hypokinetic, including the apex, while in PE the apex usually appears hyperkinetic, producing McConnell’s sign. Even though this finding is considered extremely specific for PE, it can also be observed rarely in cases of RVMI. D-sign and right-ventricular thrombus may also seldom appear in patients with right-ventricular ischemia. Therefore, the distinction between these two entities should be made utilizing all available evidence [90,91].

### 6.4. Cardiac Tamponade

FoCUS represents a critical diagnostic tool in the differential evaluation of patients presenting to the Emergency Department with chest pain and undifferentiated shock, as it allows rapid identification of signs suggestive of cardiac tamponade [92]. Clinical assessment alone is insufficient to reliably establish the diagnosis, as the classic components of Beck’s triad demonstrate limited sensitivity. In an Emergency Department-based study, hypotension, jugular venous distension, and muffled heart sounds showed sensitivities of 37.5%, 12.5%, and 37.5%, respectively, while none of the patients simultaneously exhibited all three elements of the triad [93]. On the contrary, recent evidence suggests that the application of FoCUS allows the accurate identification of the type of shock with high sensitivity and specificity with regard to obstructive shock, including cardiac tamponade [35,94]. Therefore, when clinical suspicion for tamponade exists, prompt bedside cardiac ultrasound evaluation is warranted.

FoCUS in the Emergency Department has proven to be a highly reliable modality for the detection of pericardial effusion [84]. Beyond the visualization of pericardial fluid, several echocardiographic findings are suggestive of tamponade physiology. Diastolic collapse of the right ventricle is characterized by high specificity (75–90%) but lower sensitivity (48–60%). Another early sign is systolic collapse of the right atrium, with sensitivity ranging from 50% in early tamponade to nearly 100% in advanced stages (Video S9 in the Supplementary Materials). In addition, a dilated inferior vena cava with minimal respiratory variation represents a highly sensitive finding (95–97%). Finally, marked respiratory variation in transmitral and transtricuspid inflow velocities can be observed, serving as an echocardiographic surrogate of pulsus paradoxus, which has an estimated sensitivity of approximately 82% [95]. Interestingly, application of FoCUS may provide therapeutic guidance and expedite pericardiocentesis, while reducing associated complications [96].

### 6.5. Acute Aortic Dissection

Acute aortic dissection is a rare but life-threatening cause of chest pain often mimicking ACS, especially when presenting with ST-elevation on the ECG. This accounts for when dissection extends into the coronary arteries, more commonly into the right coronary artery, presenting as inferior STEMI [97]. Managing these patients as patients with STEMI carries significant risk, as the administration of antithrombotic therapy may exacerbate bleeding and increase mortality. In a retrospective study, approximately 20% of patients with type A dissection received preoperative antithrombotic therapy, resulting in increased in-hospital mortality [98].

**Figure 9:**
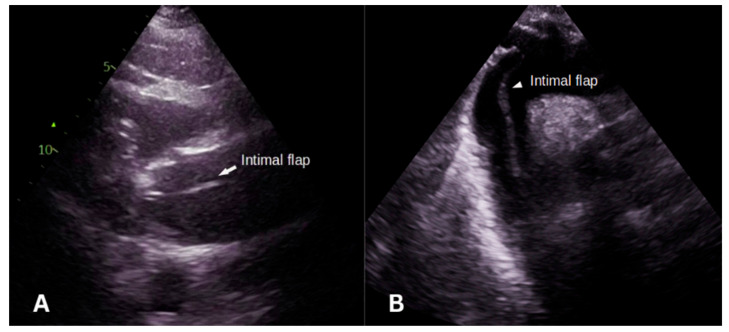
Acute aortic dissection with intimal flap (white arrow). (**A**) Parasternal long axis view; (**B**) suprasternal view of aortic arch (image courtesy of E. Kiouri and E. Polyzogopoulou).

FoCUS, enhanced by advanced modalities, may make a valuable contribution to the differential diagnosis between acute aortic dissection and ACS. Direct sonographic findings, such as visualization of an intimal flap (Figure 9A,B and Video S10 in the Supplementary Materials), double lumen or intramural hematoma, are highly specific for the diagnosis, while color Doppler imaging allows differentiation between true and false lumens based on flow velocity patterns. In addition, indirect findings—including ascending aorta dilation, aortic root dilation > 40 mm, aortic valve regurgitation and pericardial effusion or cardiac tamponade—further support the diagnosis [99].

Prospective data demonstrate that FoCUS has high specificity (94%) for confirming type A dissection when direct findings are present, while sensitivity increases from 54% to 88% when indirect signs are identified [100]. Its diagnostic value is even greater in hemodynamically unstable patients, where the presence of direct findings demonstrates absolute specificity and positive predictive value (100%), enabling immediate life-saving clinical decisions. These findings are supported by meta-analyses confirming the high diagnostic accuracy of FoCUS for type A dissection [101]. Furthermore, the use of FoCUS performed by emergency physicians has been associated with faster diagnosis and a significant reduction in diagnostic errors in the ED [102]. Overall, FoCUS represents a critical adjunct in the differential diagnosis between acute aortic dissection and ACS, facilitating early recognition and helping to avoid potentially harmful therapeutic interventions.

**Figure 10:**
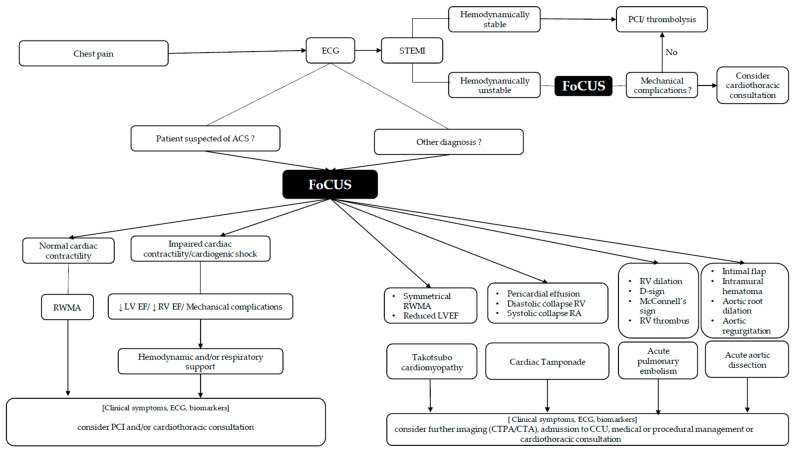
Algorithm suggesting the integration of FoCUS in patients presenting to the Emergency Department with chest pain suggestive of ACS. Abbreviations: ACS = Acute Coronary Syndrome; CTA = Computed Tomography Angiography; CCU = Coronary Care Unit; CTPA = Computed Tomography Pulmonary Angiography; ECG = electrocardiogram; EF = Ejection Fraction; LV = left ventricle; PCI = Percutaneous Coronary Intervention; RA = right atrium; RV = right ventricle; RWMAs = Regional Wall Motion Abnormalities.

Figure 10 summarizes a stepwise approach regarding the integration of FoCUS in the management of patients presenting to the Emergency Department with chest pain.

## 7. Limitations and Special Considerations

Despite its significant clinical utility as a rapid, targeted bedside tool for cardiac assessment, FoCUS has clear limitations that must be recognized during clinical application. Firstly, instrumentation used for bedside FoCUS may be of limited capabilities and/or smaller size, thus restricting image acquisition and resolution [6,22]. It is a highly operator-dependent modality, as both image acquisition and interpretation are directly influenced by the examiner’s training, experience, technical proficiency and clinical judgment [7,76,103,104]. Inadequate training is associated with reduced diagnostic sensitivity and an increased risk of misinterpretation. In addition, image quality may be significantly limited in patients with obesity, tachycardia, mechanical ventilation, or hemodynamic instability, where acquisition of adequate acoustic windows becomes challenging [7,76,103]. In such cases, a non-diagnostic or equivocal examination should not be falsely reassuring; instead, it should prompt further diagnostic evaluation. Furthermore, FoCUS should not be erroneously used as a substitute for formal echocardiography [7,105,106]. Due to its focused nature, it is designed to answer specific clinical questions, but may fail to detect significant pathologies outside the predefined diagnostic scope, such as complex valvular disease, regional wall motion abnormalities, and intracardiac masses [105,106]. Moreover, interpretation of isolated ultrasound findings without adequate clinical correlation carries the risk of inappropriate therapeutic decisions; therefore, FoCUS should function as an adjunct to clinical judgment rather than as a stand-alone diagnostic modality [76,103].

The safe implementation of FoCUS requires appropriate training, continuous supervision and clearly defined application boundaries. Operators should be aware of indications, exam expectations, limitations and contraindications [6,22,107]. Current guidelines suggest that basic competency may be achieved after a minimum of 50 supervised examinations combined with structured theoretical and hands-on training [6], whereas more advanced applications require competency-based assessment, ongoing supervision and greater clinical experience [8,104,107].

In resource-limited settings, additional barriers include equipment acquisition costs, maintenance of portable devices, limited availability of experienced trainers, high clinical workload reducing protected training time, absence of structured credentialing systems, and unclear medicolegal responsibilities [7,108,109]. Addressing these challenges requires practical and sustainable strategies, including competency-based training programs tailored to the local clinical environment, training-of-trainers models to build local educational capacity, tele-mentoring, systematic quality assurance, mandatory image archiving and clear referral pathways for comprehensive echocardiographic evaluation when findings are abnormal, equivocal or non-diagnostic [7,104,108].

## 8. Conclusions

A systematic approach to a patient suspected of ACS entails the integration of FoCUS as an auxiliary bedside tool aiming to improve diagnostic and therapeutic procedures. Diagnosis is principally based on ECG and cardiac biomarkers, yet in patients with atypical presentation of chest pain, these may be inconclusive, and FoCUS may early identify signs of diastolic dysfunction or RWMAs and reinforce the suspicion for further diagnostic investigation. On the contrary, a normal focused cardiac ultrasound examination does not exclude ACS. It should be emphasized that FoCUS serves as a complementary modality to the established diagnostic keystones, ECG and cardiac biomarkers, and cannot replace them or be employed in isolation. Additionally, in patients with ECG findings suggestive of ischemia, FoCUS may localize the ischemic region and confirm ECG abnormalities, thus identifying the culprit coronary artery. In parallel, FoCUS may detect other causes of chest discomfort presenting with ECG findings suggestive of ischemia. In patients with STEMI, it should be applied with caution so as to prevent delays to the catheterization laboratory. However, its use is mandatory in the subset of patients presenting with signs of AHF or cardiogenic shock, for the assessment of global cardiac function and the prompt recognition of AMI complications. Furthermore, its use may guide further management and the appropriate therapeutic plan, including the administration of vasoactive agents, the type of mechanical circulatory support and/or the decision to undergo invasive treatment.

Diagnostic evaluation of patients suspected of having ACS is based on the notion that time is myocardium, thus FoCUS should respect crucial time intervals and prioritize patient care according to recommended guidelines. As an adjunctive bedside tool, it may expedite diagnosis by providing valuable information on the top of ECG and cardiac biomarkers, whenever there is diagnostic uncertainty (e.g., patients in the observational zone, patients with NSTEMI/mimickers of ACS). In critical clinical scenarios, experienced operators should commit to the timely detection of signs of acute ischemia and/or identify causes of hemodynamic instability. Thus, its use may be incorporated in the initial diagnostic approach and facilitate decision-making regarding further stabilization and management. Nonetheless, FoCUS should be performed promptly to address specific diagnostic concerns and must not delay cardiac catheterization or appropriate definitive treatment.

In the emergency setting, FoCUS has an acknowledged role in the initial evaluation of patients with ACS and may expedite appropriate management. FoCUS findings should be incorporated in a holistic diagnostic and therapeutic approach, and interpretation should be done with special caution for each clinical scenario. Its targeted integration into the patient pathway to definitive treatment may improve outcomes and decrease mortality.

## Figures and Tables

**Table 1:** Possible key findings of FoCUS in the context of Acute Coronary Syndrome.

**ACS**	**Key Findings**	**Limitations**
Regional Wall Motion Abnormalities	Segmental impaired contractility	Previous MI Non-ischemic cause (focal myocarditis, Takotsubo cardiomyopathy) Cannot be used to rule out ACS
Reduced LV Ejection Fraction	“Eye ball” method Global LV impaired contractility	Chronic left-ventricular heart failure
Reduced RV Ejection Fraction	Impaired RV contractility RV dilation TAPSE < 17 mm Tricuspid valve regurgitation	Chronic right-ventricular heart failure
**ACS with Mechanical Complications**	**Key Findings**	**Limitations**
Papillary Muscle Rupture	Severe MR findings (color Doppler) Flail leaflet LV wall motion abnormalities	Chronic MR of non-ischemic cause
LV Septal Rupture	Septal wall defect (color Doppler)	B-mode alone—poor accuracy
LV Free Rupture	Pericardial effusion/Tamponade LV wall defect	

Abbreviations: ACS = Acute Coronary Syndrome; LV = left ventricle; MI = Myocardial Infarction; MR = mitral regurgitation; RV = right ventricle; TAPSE = Tricuspid Annular Plane Systolic Excursion.

## Data Availability

Data is contained within the article or the Supplementary Materials.

## Supplementary Materials

The following supporting information can be downloaded at: https://www.mdpi.com/article/10.3390/medicina62061013/s1, Videos S1–S10. Video S1 illustrates Apical 4 chamber view with apical akinesia and hypokinesia of the apical septal segment (Video courtesy S. Bezati), Video S2 illustrates Apical 4 chamber view with diffuse left ventricular hypokinesia (Video courtesy E. Kiouri), Video S3 illustrates Apical 4 chamber view with Color Doppler showing severe Mitral regurgitation (Video courtesy E. Kiouri), Video S4 illustrates 3 chamber view with Color Doppler showing severe Mitral regurgitation (Video courtesy E. Kiouri), Video S5 illustrates Apical 4 chamber view showing septal rupture; a: B-mode b: Color Doppler (Video courtesy E. Kiouri), Video S6 shows Apical 4 chamber view illustrating free left ventricular wall rupture (Video courtesy E. Polyzogopoulou), Video S7 illustrates Apical 4 chamber view with features of Takotsubo cardiomyopathy (Video courtesy E. Kiouri), Video S8 illustrates Apical 4 chamber view with dilation of the Right Ventricle and Mc Connell’s sign (Video courtesy E. Polyzogopoulou), Video S9 illustrates subxiphoid view with features of cardiac tamponade (Video courtesy E. Polyzogopoulou), Video S10 illustrates suprasternal view of the aortic arch showing acute aortic dissection (Video courtesy E. Polyzogopoulou).
