# Supplementary material for: Can FoCUS Speed Up the Management of Acute Coronary Syndrome in the Emergency Department?
Source: Medicina (Kaunas). 2026 May 23;62(6):1013. doi: 10.3390/medicina62061013 (PMC13303881; doi:10.3390/medicina62061013)
Supplement: Supplementary file 1 [file medicina-62-01013-s001.zip › Video legends.pdf]

Video S1 illustrates Apical 4 chamber view with apical akinesia and hypokinesia of the apical septal segment.

Video courtesy S.Bezati

Video S2 illustrates Apical 4 chamber view with diffuse left ventricular hypokinesia.

Video courtesy E. Kiouri

Video S3 illustrates Apical 4 chamber view Color Doppler with severe Mitral regurgitation.

Video courtesy E. Kiouri

Video S4 illustrates 3 chamber view Color Doppler with severe Mitral regurgitation.

Video courtesy E. Kiouri

Video S5 Apical 4 chamber view with Septal rupture; a: B-mode b: Color Doppler.

Video courtesy E. Kiouri

Video S6 Apical 4 chamber view illustrating free left ventricular wall rupture.

Video courtesy E.Polyzogopoulou

Video S7 illustrates Apical 4 chamber view with Takotsubo cardiomyopathy.

Video courtesy E. Kiouri

Video S8 illustrates Apical 4 chamber view with dilatation of the Right Ventricle and Mc Connell's sign.

Video courtesy E.Polyzogopoulou

Video S9 illustrates Subxiphoid view with cardiac tamponade.

Video courtesy E.Polyzogopoulou

Video S10 illustrates suprasternal view of the aortic arch with acute aortic dissection

Video courtesy E.Polyzogopoulou
